# Electronic Health Record–Integrated Legal Documentation to Measure Involuntary Mental Health Detention of Children

**DOI:** 10.1016/j.jaacop.2024.09.001

**Published:** 2024-09-18

**Authors:** Juliet Beni Edgcomb, Chi-hong Tseng, Alexandra M. Klomhaus, Ariel Seroussi, Jonathan P. Heldt, Chrislie G. Ponce, Liliana Perez, Joshua J. Lee, Bonnie T. Zima

**Affiliations:** University of California, Los Angeles, Los Angeles, California

**Keywords:** adolescent, child, electronic health records, emergencies, mental health

## Abstract

**Objective:**

To examine the prevalence and correlates of child involuntary mental health detentions through evaluation of legal documentation embedded in medical records and children’s electronic health information.

**Method:**

Medical records were analyzed from 3,440 children ages 10 to 17 years with MH-related emergency department visits in a large academic health system over 2 years (2017-2019). Bivariate analyses and random forests were deployed to identify child-, neighborhood-, and systems-level correlates of involuntary MH detentions.

**Results:**

Nearly 1 in 4 (n = 769, 22.4%) visits involved an involuntary detention. Half of detained children (n = 357, 46.4%) arrived on a detainment that was discontinued after MH provider evaluation. Odds of detention were greater among Black (odds ratio 1.33 [95% CI 1.02-1.73]) and publicly insured (odds ratio 1.63 [95% CI 1.37-1.94]) children. Children detained in prehospital settings resided in census tracts with greater social vulnerability scores (χ^2^ 13.42, *p* < .001). Machine learning classifiers (area under the curve 0.83, [95% CI 0.81-0.84]) revealed that strongest indicators of detainment included psychiatric chief concern, prior year psychiatric hospitalization, Social Vulnerability Index, and *ICD-10-CM* code for suicide or self-harm.

**Conclusion:**

Medical record–embedded legal documentation supports transparency in the use of detentions, which are common and jointly predicted by children’s clinical need and social vulnerability.

## Plain language summary

Study finds that clinical need and social vulnerability jointly predict child involuntary mental health detention. Linking electronic-format detainment orders to emergency department (ED) health records within a large urban health system in Los Angeles, the team discovered factors associated with detainment including suicide-related diagnoses, use of antipsychotic medications, history of psychiatric hospitalizations, child’s age, location of the ED visit, and neighborhood social vulnerability. Findings highlight the growing role of informatics in understanding critical emergency care processes in child psychiatry.

Child psychiatric emergencies, which often include involuntary detainment, are a leading cause of pediatric morbidity and mortality.^1^^,^^2^ Emergency mental health (MH) detainment (also called an involuntary hold or 72-hour hold) is the involuntary detention of a person presumed to be gravely disabled due to a mental illness or a danger to themselves or others.^3^ Despite calls to expand US MH crisis service capacity,^4^ the mean increase in state rates of involuntary psychiatric detentions was 3 times greater than the mean increase in state population across 25 states between 2011 and 2018.^5^ The most prevalent reason for detention is danger to self or others, and the most common maximum duration is 72 hours with a range between 23 hours and 10 days.^3^^,^^6^ Detainments do not always lead to a psychiatric hospitalization, and decision making to continue detainments or convert to voluntary status may vary by the subspecialty training of the psychiatrist.^6^ For children, national and state laws vary on the reason and by whom a minor may be detained, detainment duration, and the rights to which the child and their parent or legal guardian are entitled.^3^ Detainment of a minor implies a parent or legal guardian was not able or willing to consent to voluntary psychiatric hospitalization.^7^

The most recent robust clinical data collection on detainments of minors in the United States ended in 2003,^8^ predating widespread use of electronic health records (EHRs), *DSM-5*, and *ICD-10-CM* codes. A 2021 meta-analysis^9^ of child characteristics associated with emergency detainments across high-income countries identified only 2 US-based studies in the past 35 years.^8^^,^^10^ International studies identify increased odds of involuntary psychiatric hospitalization among children with intellectual disability,^11^ primary psychiatric diagnosis of psychosis^12^ and substance misuse,^13^ risk of harm to self or others,^14^ Black race or ethnicity,^15^ and older age.^8^^,^^16^ Although detentions account for one-fifth of psychiatric admissions among minors,^7^ surprisingly little is known about the causes, correlates, or consequences of involuntary MH detainment,^9^ or the decision to continue prehospital detainment, of children in the United States.^6^

Guided by the conceptual framework for advancing disparities research within health care systems presented by Kilbourne *et al.*,^17^ this study had a dual objective. First, we examined bivariate associations between individual characteristics documented in medical records and instances of involuntary detainment. This step aligned with the initial phase of Kilbourne’s framework, which focuses on the basic detection of populations vulnerable to detainment. Subsequently, we assessed the relative importance of child-level clinical and sociodemographic factors, neighborhood-level factors, and systems-level characteristics in predicting the use and continuation of detainment. To achieve this, we employed a translational machine learning approach capable of handling nonlinear and intersecting relations in high-dimensional data. This approach aligned with the second phase of Kilbourne’s framework, which emphasizes understanding multilevel factors that may underlie disparities in detainment. Study findings aim to inform the future development and implementation of interventions designed to precisely target equity, influence policy, and enhance the quality of care for children in emergency settings.

## Method

### Design

We conducted an observational study using EHRs of children ages 10 to 17 years. The index visit was the most recent MH-related emergency department (ED) visit for each child occurring between 2017 and 2019. Outcomes were any exposure to involuntary MH detainment (vs voluntary status), including detainments initiated in the ED and initiated in the field before hospital arrival, and among children with exposure to detainment in the ED, detainment following MH provider evaluation (vs discontinued detainment). The latter outcome was purposefully selected to distinguish risk factors for detainment supported by an ED MH provider vs detainment not supported by an ED MH provider. Independent variables were child-level clinical and sociodemographic characteristics, neighborhood-level characteristics, and systems-level characteristics.

### Data Source and Setting

The data source was medical records from a large academic health care system in urban Los Angeles County serving 4.7 million patients including more than 400,000 children (52.8% male; 0.3% American Indian or Alaska Native, 11.5% Asian, 5.7% Black, 0.2% Native Hawaiian, 43.0% White, 35.1% other race, and 23.8% Hispanic or Latino) residing in more than 1,100 census tracts. Study sites were 2 EDs, one in an academic medical center and one in a community hospital. The academic medical center ED is located within a quaternary-care teaching hospital with a colocated children’s hospital and psychiatric hospital with 3 inpatient units serving children with mental illness. The ED is staffed 24/7 with psychiatry residents and child and adolescent psychiatry fellows supervised by board-certified child and adolescent psychiatrists. The community hospital ED is affiliated with a 25-bed general pediatric ward. At this ED, children with acute MH needs are evaluated by ED physicians and licensed clinical social workers, with consultant child and adolescent psychiatrists available on an on-call basis.

The health care system was selected because it was one of the first nationally to implement EHR-integrated documentation of detainment (e5585), enabling linkage of the verbatim text of detainment and advisement documentation with health records. Since June 2016, all MH providers within the health care system use electronic format detainments.

The process for involuntary detainment of children in California, often referred to as Welfare and Institutions Code Section 5585 (WIC 5585), involves initiation by an authorized individual, who upon probable cause, may take the minor, or cause them to be taken, into custody and place them in a county-designated facility for 72-hour treatment and evaluation. The facility is required to make an application in writing stating the circumstance under which the minor’s condition was present and make “every effort to notify the minor’s parent or legal guardian as soon as possible after the minor is detained.”^18^ California laws are consistent with 9 states regarding reason for commitment and 22 states regarding duration of emergency hold.^3^

### Sampling

Visits were restricted to the most recent MH-related ED visit by each child, occurring October 1, 2017, through October 1, 2019. Dates were selected to correspond to adoption of *ICD-10-CM* and before the onset of the COVID-19 pandemic. Visits were considered MH related if associated with any of the following:

1. MH-related diagnosis in any field
2. MH-related chief concern
3. Triage screening nurse affirmed response to the question “Does this patient have a primary psychiatric concern or suspicion of psychiatric illness?”
4. MH detainment order

**Figure 1:**
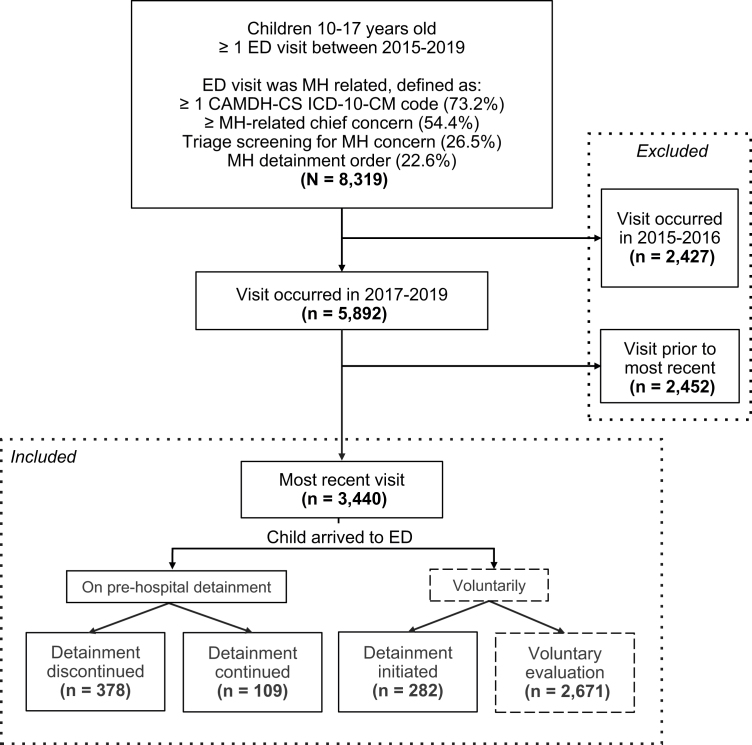
Flowchart for Study Inclusion ***Note:****Detainment refers to involuntary MH detainment of a minor (ie, 5,585 application, 72-hour hold order). Visits were restricted to 2017-2019 to correspond to adoption of electronic format MH detainment forms. ED = emergency department; CAMDH-CS = Child and Adolescent Mental Health Disorders Classification System; MH = mental health.*

The flowchart for study inclusion is presented in Figure 1.

### Study Variable Construction

We ascertained the detainment outcome by noting whether there was an order indicating arrival at the ED on a detainment (“arrived on 72 hold”) or a new detainment initiated during the ED visit (“72-hour hold order”). Initiation or continuation of detainment was determined by the presence of an electronic-format detainment note signed by an ED MH provider.

Clinical characteristics included diagnostic or billing codes, chief concerns, prior care use, medications, and laboratory tests. High cardinality features were binned to mitigate noise. We categorized *ICD-10-CM* codes by the Child and Adolescent Mental Health Disorders Classification System (CAMHD-CS) into 23 diagnostic categories.^19^ We categorized chief concerns as psychiatric vs nonpsychiatric and suicide related vs non–suicide related. Prior care use was defined as presence of ≥1 ED visits, general medical hospitalizations, or psychiatric hospitalizations within the prior 90, 180, and 365 days. Psychotropic medications received during the ED visit were consolidated using Anatomical Therapeutic Chemical classes into 8 categories (antidepressants, antiepileptics, antihistamines, antipsychotics, anxiolytics, hypnotics and sedatives, lithium, and psychostimulants). Laboratory tests related to overdose (serum acetaminophen >10 μg/mL, salicylates >3.0 mg/dL, benzodiazepines >0 ng/L, and tricyclics >3.0 ng/mL), urine drug screen results (positive), and serum alcohol (>15 mg/dL) were encoded to binary variables. ED disposition was also recorded, including discharge, general medical hospitalization, and psychiatric hospitalization (within health system or transferred). The year of ED visit, site (academic, community), child’s vital status (living, deceased), and primary ED provider (specialty, gender) were also included. Child sociodemographic characteristics included age at visit, legal sex, race (American Indian or Alaska Native, Asian, Black or African American, Native Hawaiian or Other Pacific Islander, White, or other), ethnicity (Hispanic/Latino), primary language (English, Spanish, other), and insurance status (public, private, or other/unknown).

The census-tract level Federal Information Processing System code of each child’s home address was linked to 2 established indicators of neighborhood vulnerability: the Centers for Disease Control and Prevention and Agency for Toxic Substances and Disease Registry Social Vulnerability Index (SVI)^20^ and the Health Resources and Services Administration Area Deprivation Index (ADI). The SVI is an established^21^, ^22^, ^23^, ^24^ composite index derived from 5-year American Community Survey data and indicates the relative vulnerability of every US census tract. The SVI ranks the tracts on 16 social factors, including unemployment, disability, and racial and ethnic minority status, and groups them into 4 related subdomains: socioeconomic status (eg, ≤150% poverty level, housing cost burden), household composition and disability (eg, single-parent households), minority status and language (eg, percentage Hispanic or Latino), housing type and transportation (eg, multiunit structures, no vehicle). The SVI total and subdomain scores range from 0 to 1 with higher scores representing higher vulnerability. The ADI is a ranked metric of neighborhood socioeconomic disadvantage of a census block group^25^^,^^26^ at the state (decline) and national (percentile) level.

### Analysis

We approach the analysis in 2 phases. First, we conducted bivariate analyses to compare the odds of each outcome (detainment and continuation) by levels of each characteristic (eg, sex). Second, we trained and cross-validated random forests to predict each outcome, jointly considering child-, neighborhood-, and systems-level variables (159 variables), then examined the relative feature importance of each variable. Second, we trained and cross-validated random forests to predict each outcome, jointly considering child-, neighborhood-, and systems-level variables (159 variables), then examined the relative feature importance of each variable.

The odds ratio (OR) and corresponding 95% CI were computed by child characteristics. For each OR, the comparator group was children without the specified characteristic or specified reference group. In cases where any cell had a count less than 5, Fisher exact tests were used to calculate CI. With a sample size of 3,440, we estimated at least 19 subjects per subgroup to have 80% power to detect an OR of 1.3 between groups at α = .05. Nonparametric Kruskal-Wallis tests robust to skew were used to compare median differences for each neighborhood-level characteristic.

Random forest was selected for robustness to multicollinearity, achieved through averaging of independent decision trees, and capacity to include a large number of features by assigning importance scores to variables based on contribution to model predictions.^27^ By averaging multiple decision trees in an ensemble learning approach, random forest classifiers help reduce overfitting and are less susceptible to noise and outliers in the data.^27^ For each fold, each classifier was trained with the data elements (features) and the outcome of interest. Classifiers were trained and tested using nested 10-fold cross-validation.^28^ The maximum depth (3, 5, 10, none), maximum features (sqrt, log2, and none), minimum leaf samples (1-3), minimum split (2, 5, 10), and n estimators (100-300) were tuned using nested cross-validation. Within each fold, tuning occurred exclusively on training data, and no test data were used to tune the classifier. Class weights were adjusted to be inversely proportional to the number of samples in each class (balanced). GridSearchCV was used to evaluate all hyperparameter combinations, and balanced class weights were used.^29^ Fit metrics (sensitivity, specificity, accuracy, and area under the curve [AUC]) were measured via cross-validation and reported only on test data. We calculated CIs by examining the variation across test sets.^30^ The receiver operating characteristic curve of each model was inspected to illustrate the ability of each binary classifier to differentiate the outcome of interest as its discrimination threshold is varied.^31^ SHapley Additive exPlanations (SHAP) summary plots were inspected to ascertain SHAP values for feature importances.^32^ To explore variability performance and feature importance across classifier types, lasso logistic regression classifiers^33^ were also developed, and performance (accuracy, AUC) was compared with the random forest classifiers.

Analyses were conducted in Python (version 3.12; Python Software Foundation, Wilmington, Delaware) with scikit-learn (version 1.2.2) toolboxes, sklearn.linear_model.LogisticRegression, sklearn.linear_model.lasso, sklearn.model_selection.GridSearchCV, and sklearn.metrics. Code is available from the authors upon request.

## Results

The sample consisted of 3,440 ED visits by unique children (Table 1). Of these children, 769 (22.4%) were detained and 2,671 (77.6%) were not detained. Of children detained, 378 (49.2%) of detainments were started in the prehospital setting and discontinued, 109 (14.1%) of detainments were started in the prehospital setting and continued, and 282 (36.7%) were newly initiated after MH provider evaluation. The sample contained information on 110 unique MH providers.

**Table 1:** Variation in Child Characteristics by Odds of Mental Health Detainment Use and Continuation

Characteristics	n	(%)	Detainment vs voluntary		Detainment continued/initiated vs discontinued	
			OR	(95% CI)	OR	(95% CI)
Total	3,440	(100)				
Sex						
Male	1,608	(46.7)	1.04	(0.88-1.22)	1.12	(0.84-1.49)
Female	1,831	(53.2)	0.96	(0.81-1.13)	0.89	(0.67-1.19)
Age group, y						
10-12.9	710	(20.6)	Reference		Reference	
13-15.9	1,334	(38.8)	1.16	(0.93-1.45)	1.53	(1.02-2.29)
16-17.9	1,396	(40.6)	1.20	(0.96-1.50)	1.93	(1.30-2.89)
Race and ethnicity						
Not Hispanic or Latino						
American Indian or Alaska Native	9	(0.3)	0.43	(0.05-3.47)	—	
Asian	164	(4.8)	1.29	(0.90-1.84)	1.06	(0.58-1.95)
Black or African American	310	(9)	1.33	(1.02-1.73)	1.07	(0.68-1.69)
Hispanic or Latino	919	(26.7)	0.96	(0.80-1.15)	0.64	(0.47-0.89)
Multiple races	87	(2.5)	1.18	(0.70-2.00)	0.70	(0.28-1.75)
Native Hawaiian or other Pacific Islander	5	(0.1)	2.23	(0.39-13.90)	0.97	(0.01-76.03)
White	1,715	(49.9)	0.93	(0.79-1.09)	1.33	(1.00-1.77)
Other race or ethnicity^a^	211	(6.1)	0.83	(0.58-1.18)	1.25	(0.66-2.35)
Insurance status						
Private	1,475	(42.9)	0.99	(0.84-1.16)	1.38	(1.04-1.38)
Public	916	(26.6)	1.63	(1.37-1.94)	0.90	(0.67-1.21)
Other/NA	1,187	(34.5)	0.68	(0.57-0.81)	0.83	(0.60-1.13)
Site						
Academic medical center	2,535	(73.7)	11.18	(7.78-16.08)	34.84	(4.73-256.57)
Community hospital	905	(26.3)	0.09	(0.06-0.13)	—	
Chief concern						
Psychiatric (including suicide-related)	1,870	(54.4)	7.22	(5.82-8.96)	2.19	(1.43-3.33)
Suicide-related	635	(18.5)	3.97	(3.30-4.78)	1.31	(0.98-1.76)
ED diagnostic code category (CAMHD-CS)^b^						
ADHD	742	(21.6)	1.51	(1.26-1.82)	1.46	(1.06-2.00)
Anxiety disorders	1,075	(31.3)	1.27	(1.07-1.51)	1.22	(0.91-1.64)
Autism spectrum disorder	395	(11.5)	1.34	(1.05-1.70)	1.22	(0.81-1.84)
Bipolar and related disorders	157	(4.6)	2.97	(2.15-4.12)	1.97	(1.17-3.29)
Communication disorders	18	(0.5)	0.99	(0.33-3.02)	0.97	(0.14-6.90)
Depressive disorders	1,309	(38.1)	2.43	(2.06-2.86)	1.21	(0.91-1.60)
Developmental delay or unspecified neurodevelopmental disorder	67	(1.9)	0.75	(0.40-1.42)	0.69	(0.22-2.18)
Disruptive, impulse control, and conduct disorders	203	(5.9)	4.06	(3.05-5.42)	1.58	(1.04-2.41)
Feeding and eating disorders	102	(3)	0.64	(0.37-1.10)	0.43	(0.15-1.26)
Intellectual disability	57	(1.7)	0.83	(0.43-1.61)	4.43	(0.95-20.64)
Mental health symptom	454	(13.2)	3.27	(2.66-4.02)	1.48	(1.07-2.05)
Miscellaneous	170	(4.9)	0.90	(0.61-1.31)	1.02	(0.52-2.02)
Motor disorders	38	(1.1)	1.82	(0.93-3.57)	0.60	(0.19-1.85)
Neurocognitive disorders	61	(1.8)	0.12	(0.03-0.47)	—	
Obsessive-compulsive and related disorders	160	(4.7)	1.47	(1.04-2.09)	1.21	(0.67-2.19)
Personality disorders	23	(0.7)	1.23	(0.48-3.12)	1.94	(0.35-10.67)
Schizophrenia spectrum and other psychotic disorders	130	(3.8)	2.49	(1.74-3.57)	2.61	(1.41-4.82)
Sexuality and gender identity disorders	33	(1)	2.59	(1.29-5.19)	1.30	(0.45-3.77)
Specific learning disorders	12	(0.3)	1.74	(0.52-5.80)	0.97	(0.14-6.90)
Substance-related and addictive disorders	458	(13.3)	0.99	(0.79-1.26)	1.75	(1.14-2.69)
Suicide or self-injury	1,170	(34)	3.96	(3.35-4.69)	1.99	(1.49-2.67)
Trauma and stressor-related disorders	209	(6.1)	2.39	(1.79-3.20)	1.42	(0.89-2.25)
Psychiatric comorbidity (≥2 diagnostic groups)	2,042	(59.4)	6.76	(5.38-8.49)	4.29	(2.58-7.13)
Acute care use						
Prior ED use (≥1 visit)						
Past 90 days	546	(15.9)	1.30	(1.03-1.64)	1.83	(1.37-2.45)
Past 180 days	753	(21.9)	1.45	(1.17-1.79)	0.85	(0.57-1.27)
Past 365 days	1,004	(29.2)	1.26	(1.02-1.55)	0.77	(0.53-1.11)
Prior medical hospitalization (≥1 visit)						
Past 90 days	74	(2.2)	0.11	(0.03-0.46)	0.72	(0.50-1.03)
Past 180 days	113	(3.3)	0.22	(0.09-0.53)	0.64	(0.11-3.86)
Past 365 days	174	(5.1)	0.38	(0.21-0.70)	0.47	(0.14-1.59)
Prior psychiatric hospitalization (≥1 visit)						
Past 90 days	116	(3.4)	5.73	(3.85-8.54)	0.74	(0.44-1.24)
Past 180 days	184	(5.3)	6.72	(4.83-9.36)	0.65	(0.42-0.99)
Past 365 days	242	(7)	5.72	(4.24-7.72)	0.56	(0.37-0.85)
Medications received during ED visit						
Antidepressant	689	(20)	2.82	(1.90-2.74)	3.87	(2.78-5.39)
Antiepileptic	153	(4.4)	2.79	(2.00-3.88)	3.32	(1.85-5.94)
Antihistamine	127	(3.7)	1.76	(1.20-2.57)	3.28	(1.5-6.77)
Antipsychotic	458	(13.3)	3.32	(2.71-4.08)	2.99	(2.12-4.22)
Anxiolytic	295	(8.6)	1.57	(1.20-2.40)	3.13	(1.91-5.16)
Hypnotic or sedative	62	(1.8)	1.55	(0.90-2.67)	2.13	(0.80-5.67)
Lithium	52	(1.5)	3.29	(1.90-5.70)	1.24	(0.56-2.76)
Psychostimulant	273	(7.9)	1.77	(1.35-2.31)	3.13	(1.91-5.16)
Injectable medication	110	(3.2)	2.21	(1.49-3.28)	6.29	(2.62-15.10)
Urine drug screen results (positive)^c^						
Amphetamine	71	(2.1)	1.17	(0.72-1.90)	0.56	(0.27-1.18)
Benzodiazepines	47	(1.4)	1.26	(0.70-2.26)	0.74	(0.30-1.85)
Cannabis	199	(5.8)	0.98	(0.71-1.34)	0.76	(0.45-1.29)
Cocaine	9	(0.3)	0.59	(0.09-2.80)	0.62	(0.03-37.01)
Opiates	9	(0.3)	0.59	(0.09-2.80)	0.62	(0.03-37.01)
Ethanol	42	(1.2)	0.27	(0.12-0.58)	2.21	(0.27-18.19)
Disposition						
Discharged without hospitalization^d^	2,127	(61.8)	0.37	(0.31-0.43)	0.15	(0.11-0.21)
General medical hospitalization	315	(9.2)	0.15	(0.09-0.25)	0.97	(0.18-5.22)
Psychiatric hospitalization	937	(27.2)	3.53	(2.96-4.22)	1.75	(1.30-2.34)
Within health system	706	(20.5)	2.92	(2.44-3.49)	1.49	(1.11-2.01)
Transferred outside health system	198	(5.8)	9.04	(6.61-12.36)	37.59	(15.17-93.10)

Note: ORs represent the odds of each outcome by child characteristic (eg, the odds of detainment by sex). ORs were omitted when sample size was insufficient. 95% CIs were calculated via Fisher exact tests for 2 × 2 matrices with n < 5 in any cell. ADHD = attention-deficit/hyperactivity disorder; CAMHD-CS = Child and Adolescent Mental Health Disorders Classification System; ED = emergency department; NA = not available; OR = odds ratio.

a Other includes patient refused and unknown.

b Diagnostic code groups from CAMHD-CS.

c Urine drug screen was obtained for 27.5% of sample.

d Discharged without hospitalization includes eloped (n = 4), left without being seen (n = 2), left against medical advice (n = 2), inpatient rehabilitation facility (n = 3), law enforcement (n = 1), skilled nursing (n = 1), and expired (n = 3).

Odds of detainment varied by child clinical and sociodemographic characteristics (Table 1). For each OR, the comparator group was children without the specified characteristic (raw counts and proportions are listed in Table S1, available online). Greater odds of involuntary detainment were observed among children with an MH-related chief concern (OR 7.22 [95% CI 5.82-8.96]) and among children with *ICD-10-CM* codes for bipolar disorder (OR 2.97 [95% CI 2.15-4.12]), psychosis (OR 2.49 [95% CI 1.74-3.57]), suicide or self-injury (OR 3.96 [95% CI 3.35-4.69]), and trauma and stressor-related disorders (OR 2.39 [95% CI 1.79-3.20]). Greater odds of detainment were also observed among children who received psychotropic medications in the ED, including antidepressants (OR 2.82 [95% CI 1.90-2.74]), antiepileptics (OR 2.79 [95% CI 2.00-3.88]), antipsychotics (OR 3.32 [95% CI 2.71-4.08]), lithium (OR 3.29 [95% CI 1.90-5.70]), and injectable psychotropic medications (OR 2.21 [95% CI 1.49-3.28]). Odds of detainment were much higher among children evaluated at the academic medical center site (OR 11.18 [95% CI 7.78-16.08]) and children with a prior year psychiatric hospitalization (OR 5.72-6.72). Detainments were moderately elevated among children with an ED visit in the past year (OR 1.26-1.45). Children at lower odds of a detainment had a medical hospitalization in the past year (OR 0.11-0.38), had a positive ethanol screen (OR 0.27 [95% CI 0.12-0.58]), were discharged (OR 0.37 [95% CI 0.31-0.43]) or admitted to a general medical hospital (OR 0.15 [95% CI 0.09-0.25]). Odds of detainment initiation or continuation (vs discontinuation) after MH provider evaluation were higher among children with an MH-related chief concern (OR 2.19 [95%CI 1.43-3.33]) and varied by diagnosis with highest odds of continuation among children with *ICD-10-CM* codes for bipolar disorders (OR 1.97 [95% CI 1.17-3.29]), schizophrenia (OR 2.67 [95% CI 1.41-4.82]), and suicide or self-injury (OR 1.99 [95% CI 1.49-2.67]). Greatest odds of detainment continuation were observed among children treated with antidepressants (OR 3.87 [95% CI 2.78-5.39]), antiepileptics (OR 3.32 [95% CI 1.85-5.94]), antihistamines (OR 3.28 [95% CI 1.5-6.77]), antipsychotics (OR 2.99 [95% CI 2.12-4.22]), anxiolytics (OR 3.13 [95% CI 1.91-5.16]), psychostimulants (OR 3.13 [95% CI 1.91-5.16]), and injectable medications (OR 6.29 [95% CI 2.62-15.10]).

While odds of detainment use did not differ by sex or age, Black or African American (OR 1.33 [95% CI 1.02-1.73]) children and children with public insurance (OR 1.63 [95% CI 1.37-1.94]) had greater odds of involuntary detainment compared with children of other races and children with private or unknown insurance status. Children transferred outside of the health care system also had greater odds of detainment (OR 9.04 [95% CI 6.61-12.36]) compared with children not transferred. Moreover, compared with preteens (ages 10-12), odds of detainment continuation were greater among mid-adolescents (ages 13-15) (OR 1.53 [95% CI 1.02-2.29]) and older teens (ages 16-17) (OR 1.93 [95% CI 1.30-2.89]). Odds were greater among privately insured (OR 1.38 [95% CI 1.04-1.38]) children and children with a recent MH ED visit (OR 1.83 [95% CI 1.37-2.45]. Hispanic or Latino children had lower odds of detainment continuation (OR 0.64 [95% CI 0.47-0.89]) compared with children of other ethnicities. Children transferred to another hospital had exceedingly higher odds of detainment continuation (OR 37.59 [95% CI 15.17-93.10]) compared with children not transferred.

Greatest median SVI scores were observed among children who arrived on a hold that was discontinued following MH provider evaluation, and lowest SVI scores were observed among children who were evaluated on a voluntary basis (Table 2). There was a significant association in detainment initiation and continuation by SVI total scores (median [interquartile range (IQR)]: voluntary 0.37 [0.18-0.64] vs discontinued 0.46 [0.25-0.72] vs continued 0.37 [0.18-0.64]; *p* = .001) and the SVI subdomain scores of socioeconomic status (median [IQR]: voluntary 0.28 [0.13-0.57] vs discontinued 0.38 [0.17-0.63] vs continued 0.32 [0.12-0.60]; *p* = .01), minority status and language (median [IQR]: voluntary 0.67 [0.54-0.85] vs discontinued 0.75 [0.58-0.87] vs continued 0.69 [0.56-0.85]; *p* = .003), and housing type and transportation (median [IQR]: voluntary 0.50 [0.23-0.75] vs discontinued 0.61 [0.33-0.80] vs continued 0.48 [0.22-0.77]; *p* = .002). There were no significant differences by ADI score (*p* = .36-.56).

**Table 2:** Variation in Neighborhood-Level Child Characteristics by Involuntary Mental Health Detainment Use and Continuation

Characteristic^a^	Total sample, (n = 3,440)		Voluntary (n = 2,701)		Detainment^b^				KW χ^2^	*p*^c^
					Discontinued (n = 378)		Continued/initiated (n = 391)			
	Mdn	(IQR)	Mdn	(IQR)	Mdn	(IQR)	Mdn	(IQR)		
ADI										
National (percentile)	5	(2-12)	5	(2-12)	6	(2-11)	5	(2-11)	1.17	.56
State (decile)	2	(1-5)	2	(1-5)	3	(1-5)	2	(1-4)	1.95	.36
SVI										
Socioeconomic status	0.29	(0.13-0.57)	0.28	(0.13-0.57)	0.38	(0.17-0.63)	0.32	(0.12-0.6)	9.96	.01
Household composition and disability	0.24	(0.11-0.44)	0.25	(0.11-0.45)	0.24	(0.11-0.43)	0.22	(0.11-0.44)	1.16	.56
Minority status and language	0.68	(0.55-0.85)	0.67	(0.54-0.85)	0.75	(0.58-0.87)	0.69	(0.56-0.85)	11.44	.003
Housing type and transportation	0.51	(0.24-0.75)	0.50	(0.23-0.75)	0.61	(0.33-0.80)	0.48	(0.22-0.77)	12.63	.002
Total	0.38	(0.19-0.65)	0.37	(0.18-0.64)	0.46	(0.25-0.72)	0.37	(0.18-0.64)	13.42	.001

Note: ADI = Area Deprivation Index; IQR = interquartile range; KW = Kruskal-Wallis; Mdn = median; MH = mental health; SVI = Social Vulnerability Index.

a ADI scores were missing for 14.8% (n = 446) of children, and SVI scores were missing for 26.0% (n = 779) of children. For both ADI (National ADI Scale 0-100; State ADI Scale 0-10) and SVI (Total and Subdomains Scale 0-1), lower scores indicate less vulnerability, and higher scores indicate greater vulnerability.

b Detainment status following MH provider evaluation.

c *p* values were based on KW χ^2^ (df = 2) comparing child neighborhood ADI and SVI scores across 3 mutually exclusive groups: voluntary (no MH detainment), MH detainment (discontinued) after MH provider evaluation, and MH detainment (continued/initiated) after MH provider evaluation.

**Figure 2:**
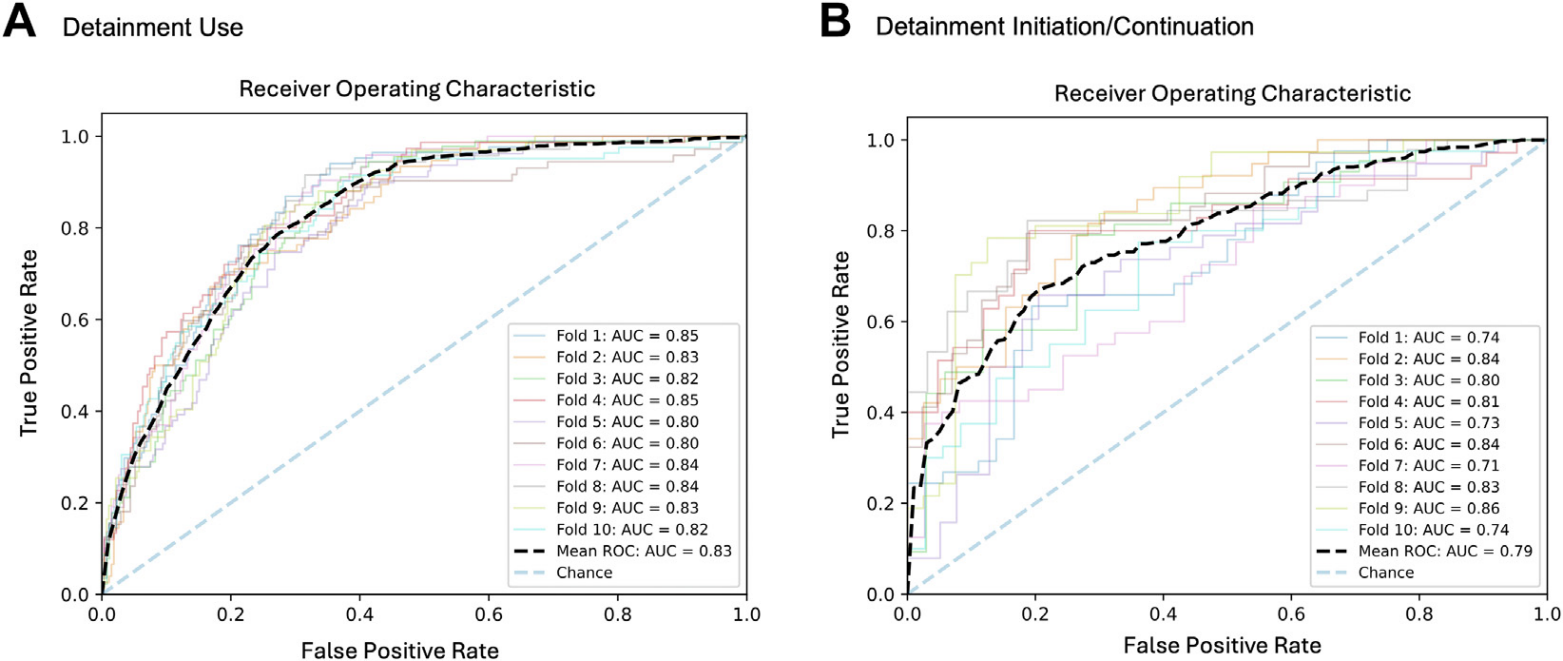
Receiver Operating Characteristic Curves ***Note:****Receiver operating characteristic curves for random forest classifiers differentiating (A) mental health detainment and (B) mental health detainment initiation or continuation following emergency department provider evaluation, with variation in performance by fold. AUC = area under the curve; ROC = receiver operating characteristic.*

**Figure 3:**
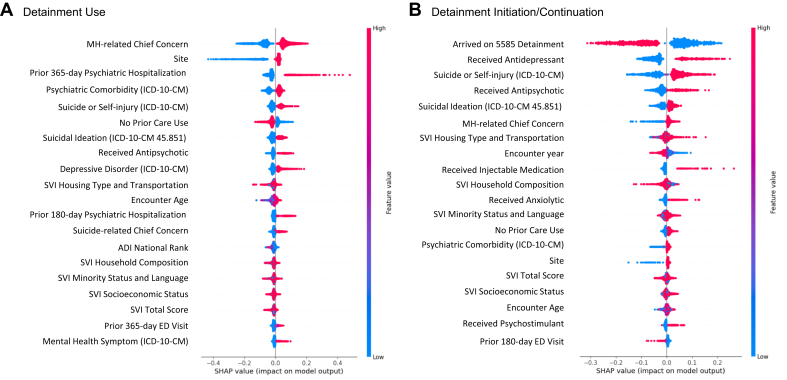
SHapley Additive exPlanations (SHAP) ***Note:****Summary plot of SHAP values from random forest classifiers differentiating (A) mental health detainment and (B) mental health detainment initiation or continuation following emergency department provider evaluation, with variation in performance by fold. ADI = Area Deprivation Index; ED = emergency department; MH = mental health; SVI = Social Vulnerability Index. Please note color figures are available online.*

Random forest classifiers differentiated involuntary detainment (vs voluntary status) with good discriminative performance (accuracy 0.80 [95% CI 0.78-0.81] and AUC 0.83 [95% CI 0.82-0.84]) (Figure 2, Figure 3; Table S2, available online). Features of highest importance included child-level clinical (MH-related chief concern, prior psychiatric hospitalization, *ICD* code for suicide and self-harm, receipt of an antipsychotic medication), child-level sociodemographic (encounter age), neighborhood-level (SVI scores and ADI national ranking), and systems-level (site) characteristics. Among detained children, detainment continuation (vs discontinuation) was differentiated with accuracy 0.72 (95% CI 0.69-0.76) and AUC 0.80 (95% CI 0.77-0.83). Features of highest importance included child-level clinical (receipt of an antidepressant, antipsychotic, or injectable medication, *ICD* code for suicide and self-harm), neighborhood-level (SVI scores), and systems-level (arrival on a 5585 detainment, site) characteristics. Lasso logistic regression classifiers performed similarly to random forest classifiers in classification of detainment (accuracy 0.81 [95% CI 0.80-0.82], AUC 0.83 [95% CI 0.81-0.84]) and continuation (accuracy 0.71 [95% CI 0.68-0.74], AUC 0.79 [95% CI 0.76-0.82]).

## Discussion

This study characterized involuntary MH detentions among minors and classified the use and continuation of detainments using EHR data. More than 1 in 5 children with an MH-related ED visit were involuntarily detained, and half of detainments were continued after psychiatric evaluation. Both bivariate analyses and translational machine learning pointed to the joint role of perceived clinical need and contextual factors, including history of psychiatric hospitalization, suicide-related diagnosis, antipsychotic medications, age, site, and SVI. Study findings underscore multilevel determinants of involuntary detainment and the interaction of detainment with concurrent and subsequent clinical care processes.

Clinical need predicted detainment use and continuation. Odds of detainment were 2 to 3 times higher among children with major mood and psychotic disorders and suicide-related diagnoses compared with children without these diagnoses. There was greater use of detainments among children with psychiatric comorbidity, children with prior psychiatric hospitalizations, and children who were administered psychotropic medications in the ED (particularly antipsychotics, lithium, and injectable psychotropic medications) compared with children with a single MH diagnosis, children with no prior hospitalizations, and children who did not receive psychotropic medications. MH providers continued or initiated detainments more than twice as often in children with psychotic disorders, 4 times as often in children with psychiatric comorbidity, and 6 times as often in children receiving an injectable psychotropic medication compared with children without these disorders. These findings align with recent international studies that have described higher odds of detainment among children with psychosis^15^ and risk of harm to self.^34^ Although previous non-US samples have identified substance misuse as predictive of involuntary psychiatric hospitalization,^13^ in this sample, serum alcohol level was positively associated with voluntary status, and there were no significant differences in detainment use by substance-related diagnosis. Moreover, while previous studies describe older age as a risk factor for involuntary psychiatric hospitalization,^13^ this study found that older adolescents were at increased odds for detainment continuation but not at increased odds of detainment overall. Findings support a difference in populations affected by detainment in emergency settings compared with detainment during psychiatric hospitalization.^6^ Most studies ended data collection before widespread adoption of EHRs and only a handful (in the Netherlands,^14^ United Kingdom,^35^ and Belgium^36^) occurred in the last 10 years. Nuances in clinical drivers of detainment may reflect variation in national laws related to detainments, changes in data collection methodology, and evolution of diagnostic code structures and practices over time.

Study findings further suggest that multilevel intersecting variables, beyond clinical need, are associated with the use and continuation of detainments. At the individual level, child race and ethnicity emerged as significant predictors of detainment. Black and African American children experienced higher odds (1.3×) of involuntary detention and Hispanic and Latino children had lower odds of prehospital detainment continuation (0.64×) compared with children of other races and ethnicities. Findings add to 2 early single-site studies^8^^,^^10^ that identified racial and ethnic differences in use of MH detainments. Of note, race and ethnicity did not emerge as variables with high individual feature importance in multivariate analyses. However, these bivariate associations may reflect common underlying risk factors that did emerge as highly important, such as diagnosis of a depressive disorder^37^ and receipt of antipsychotic medication,^38^ which may contribute to perceived dangerousness to self or others. Children with public insurance were more likely to be detained compared with children with private or unknown insurance status, but providers were more likely to continue detainments of privately insured children. Thus, these disparities may be conceptualized within the broader context of sociostructural factors, including systemic racism, socioeconomic inequities, and differential access to health care resources, which may influence both the likelihood of detainment and the perceived clinical characteristics at the time of detention and ED evaluation. Further, as our results are associative, the directionality of the association between detainment and clinical characteristics is unclear. For example, a child detained may be more likely to receive an injectable psychotropic medication on arrival to the ED, and a child who receives an injectable antipsychotic may be more likely to be subsequently detained.

At the neighborhood level, we observed greatest median SVI among children who arrived at the ED already detained and for whom the detainment was discontinued once the child was evaluated by a MH provider. Discontinued detainments occurred most often among children from neighborhoods with lower socioeconomic status, greater density of racial and ethnic minority populations, and more vulnerable housing (multiunit structures, crowding, no vehicle access, and group quarters). When other services are inaccessible or unavailable, children enter MH care through crisis services, including ED visits, psychiatric emergency response teams, and police contact.^10^ Use of prehospital detainments later determined to not meet criteria by an MH provider may reflect contact of socially vulnerable children with crisis MH services and police.^39^ In multivariate classification models, SVI total and subdomains were retained as features of high classification performance alongside clinical characteristics, but variation in directionality of the association by subdomain suggested the role of more granular indicators of contextual vulnerability beyond composite indices. To our knowledge, this is the first US-based study associating involuntary MH detainment use among minors with neighborhood-level metrics and integrating clinical data. While children are frequently detained in the prehospital setting, the risk factors for detainment in a field crisis setting may differ compared with children detained after MH provider evaluation. Given that arrival on a detainment was a strong predictor of MH provider decision making to detain the child, future work is warranted to further evaluate drivers of and disparities in use of prehospital detainments.

At a systems level, findings support the potential of EHR-embedded detainment orders to inform policy surrounding the involuntary MH detainment of minors. For example, compared with individual patient characteristics, there were exceedingly higher odds that a child was continued on a detainment when transferred to an outside facility, raising questions of whether a continued detainment is clinically indicated or used to comply with policy related to safe transfer of patients. Further, ED site emerged as an important feature in classifying detainment use and continuation, suggesting the role of care setting in determining clinical decision making regarding detainments. Variation by site may also reflect the lack of standardized clinical protocols and treatment guidelines on use of involuntary MH detainments in children. Future work is needed to measure MH outcomes associated with detainment policies at the system, county, and state levels. This linkage would support greater precision of safety interventions and quality of emergency care.

Limitations of this study include focus on a single health care system, reliance on indicators readily encoded in structured data, and omission of variables with high degree of missingness. The sample may not generalize to less resourced settings, such as safety-net hospitals. Provider information was available only from electronic format detainments, and thus it was not possible to determine the initiating party of prehospital detainments recorded in paper forms. Information contained in the medical record is limited by biases and idiosyncrasies of the clinical documenter and thus may imperfectly reflect the reality of the clinical scenario. Going forward, multisite validation together with geographic linkage of data from detainments with granular publicly available data (eg, police activity) could more precisely identify social determinants driving children’s vulnerability to detainment.

This is one of the first studies in the United States to leverage electronic documentation of MH detainments and link detainment to information captured in medical records. Findings reinforce urgent calls^40^^,^^41^ to address disparities in acute child MH care and build capacity to deliver high-quality care in emergency settings. Joint consideration of multilevel determinants is necessary to understand clinical decision making regarding detainment and how detainment interfaces with complex clinical trajectories of access and quality of child MH care.

## CRediT authorship contribution statement

**Juliet Beni Edgcomb:** Writing – review & editing, Writing – original draft, Software, Project administration, Methodology, Investigation, Funding acquisition, Formal analysis, Data curation, Conceptualization. **Chi-hong Tseng:** Writing – review & editing, Supervision, Software, Methodology, Formal analysis, Data curation. **Alexandra M. Klomhaus:** Writing – review & editing, Software, Methodology, Investigation, Formal analysis, Data curation. **Ariel Seroussi:** Writing – review & editing, Software, Methodology. **Jonathan P. Heldt:** Writing – review & editing, Supervision, Software, Methodology. **Chrislie G. Ponce:** Writing – review & editing, Project administration, Methodology, Investigation, Data curation. **Liliana Perez:** Writing – review & editing, Project administration, Methodology, Investigation, Data curation. **Joshua J. Lee:** Writing – review & editing, Software, Methodology, Investigation, Formal analysis, Data curation. **Bonnie T. Zima:** Writing – original draft, Supervision, Resources, Methodology, Investigation, Funding acquisition, Conceptualization, Writing – review & editing.
