# Supplementary material for: Electronic Health Record–Integrated Legal Documentation to Measure Involuntary Mental Health Detention of Children
Source: JAACAP Open. 2024 Sep 18;3(3):689–700. doi: 10.1016/j.jaacop.2024.09.001 (PMC12414314; doi:10.1016/j.jaacop.2024.09.001)
Supplement: Supplemental 1 [file mmc1.docx]

**Supplement**

**Table S1.** Counts of children by sociodemographic, clinical, and prior care use characteristics, by legal status with Odds Ratios (95% Confidence Intervals) for the use of involuntary detainment and, among those detained, initiation or continuation vs discontinuation after psychiatrist evaluation.

**Table S2.** Classifier performance metrics and contingency matrix for detainment use and continuation.

| **Table S1.** Counts of children by sociodemographic, clinical, and prior care use characteristics, by legal status with Odds Ratios (95% Confidence Intervals) for the use of involuntary detainment and, among those detained, initiation or continuation vs discontinuation after psychiatrist evaluation. | | | | | | | | | | | | |
| --- | --- | --- | --- | --- | --- | --- | --- | --- | --- | --- | --- | --- |
|  | Study Sample | | Voluntary | | Any Involuntary MH Detainment | | MH detainment discontinued after provider evaluation | | MH detainment continued or initiated after provider evaluation | | Any MH detainment vs. voluntary | MH detainment continued or initiated vs. discontinued after provider evaluation |
|  | n | % | n | % | n | % | n | % | n | % | OR (95%CI) | OR (95%CI) |
| Total | 3440 |  | 2670 | 77.6 | 769 | 22.3 | 378 |  | 391 |  |  |  |
| Sex |  |  |  |  |  |  |  |  |  |  |  |  |
| Male | 1608 | 46.7 | 1243 | 46.0 | 365 | 47.5 | 174 | 46.0 | 191 | 48.8 | 1.04 (0.88-1.22) | 1.12 (0.84-1.49) |
| Female | 1831 | 53.2 | 1427 | 52.8 | 404 | 52.5 | 204 | 54.0 | 200 | 51.2 | 0.96 (0.81-1.13) | 0.89 (0.67-1.19) |
| Age group, y |  |  |  |  |  |  |  |  |  |  |  |  |
| 10 - 12.9 | 710 | 20.6 | 567 | 21.0 | 143 | 18.6 | 86 | 22.8 | 57 | 14.6 | ref | ref |
| 13 - 15.9 | 1334 | 38.8 | 1032 | 38.2 | 302 | 39.3 | 150 | 39.7 | 152 | 38.9 | 1.16 (0.93-1.45) | 1.53 (1.02-2.29) |
| 16 - 17.9 | 1396 | 40.6 | 1072 | 39.7 | 324 | 42.1 | 142 | 37.6 | 182 | 46.5 | 1.20 (0.96-1.50) | 1.93 (1.30-2.89) |
| Race and ethnicity^a^ |  |  |  |  |  |  |  |  |  |  |  |  |
| Not Hispanic or Latino |  |  |  |  |  |  |  |  |  |  |  |  |
| American Indian or Alaska Native | 9 | 0.3 | 8 | 0.3 | 1 | 0.1 | 1 | 0.3 | 0 | 0.0 | 0.43 (0.01-3.24) | - |
| Asian | 164 | 4.8 | 120 | 4.4 | 44 | 5.7 | 21 | 5.6 | 23 | 5.9 | 1.29 (0.90-1.84) | 1.06 (0.58-1.95) |
| Black or African American | 310 | 9.0 | 226 | 8.4 | 84 | 10.9 | 40 | 10.6 | 44 | 11.3 | 1.33 (1.02-1.73) | 1.07 (0.68-1.69) |
| Multiple Races | 87 | 2.5 | 56 | 2.1 | 19 | 2.5 | 11 | 2.9 | 8 | 2.0 | 1.18 (0.70-2.00) | 0.70 (0.28-1.75) |
| Native Hawaiian or Other Pacific Islander | 5 | 0.1 | 3 | 0.1 | 2 | 0.3 | 1 | 0.3 | 1 | 0.3 | 2.32 (0.19-20.23) | 0.97 (0.01-76.03) |
| White | 1715 | 49.9 | 1343 | 49.7 | 372 | 48.4 | 169 | 44.7 | 203 | 51.9 | 0.93 (0.79-1.09) | 1.33 (1.00-1.77) |
| Other | 211 | 6.1 | 170 | 6.3 | 41 | 5.3 | 18 | 4.8 | 23 | 5.9 | 0.83 (0.58-1.18) | 1.25 (0.66-2.35) |
| Hispanic or Latino | 919 | 26.7 | 718 | 26.6 | 201 | 26.1 | 115 | 30.4 | 86 | 22.0 | 0.96 (0.80-1.15) | 0.64 (0.47-0.89) |
| Insurance Status |  |  |  |  |  |  |  |  |  |  |  |  |
| Private | 1475 | 42.9 | 1147 | 42.5 | 328 | 42.7 | 146 | 38.6 | 182 | 46.5 | 0.99 (0.84-1.16) | 1.38 (1.04-1.38) |
| Public | 916 | 26.6 | 651 | 24.1 | 265 | 34.5 | 135 | 35.7 | 130 | 33.2 | 1.63 (1.37-1.94) | 0.90 (0.67-1.21) |
| Other/NA | 1187 | 34.5 | 972 | 36.0 | 215 | 28.0 | 113 | 29.9 | 102 | 26.1 | 0.68 (0.57-0.81) | 0.83 (0.60-1.13) |
| Site |  |  |  |  |  |  |  |  |  |  |  |  |
| Academic medical center | 2535 | 73.7 | 1798 | 66.6 | 737 | 95.8 | 347 | 91.8 | 390 | 99.7 | 11.18 (7.78-16.08) | 34.84 (4.73-256.57) |
| Community hospital | 905 | 26.3 | 873 | 32.3 | 32 | 4.2 | 31 | 8.2 | 1 | 0.3 | 0.09 (0.06-0.13) | 0.03 (0.0007-0.17) |
| Chief complaint |  |  |  |  |  |  |  |  |  |  |  |  |
| Any psychiatric, including suicide-related | 1870 | 54.4 | 1211 | 44.8 | 659 | 85.7 | 306 | 81.0 | 353 | 90.3 | 7.22 (5.82-8.96) | 2.19 (1.43-3.33) |
| Suicide-related | 635 | 18.5 | 348 | 12.9 | 287 | 37.3 | 129 | 34.1 | 158 | 40.4 | 3.97 (3.30-4.78) | 1.31 (0.98-1.76) |
| ED ICD-10-CM Diagnosis^b^ |  |  |  |  |  |  |  |  |  |  |  |  |
| Accidental or Undetermined Poisoning | 25 | 0.7 | 25.0 | 0.9 | 0 | 0.0 | 0 | 0.0 | 0 | 0.0 | - | - |
| ADHD | 742 | 21.6 | 532.0 | 19.7 | 210 | 27.3 | 89 | 23.5 | 121 | 30.9 | 1.51 (1.26-1.82) | 1.46 (1.06-2.00) |
| Anxiety Disorders | 1075 | 31.3 | 803.0 | 29.7 | 272 | 35.4 | 125 | 33.1 | 147 | 37.6 | 1.27 (1.07-1.51) | 1.22 (0.91-1.64) |
| Autism Spectrum Disorder | 395 | 11.5 | 288.0 | 10.7 | 107 | 13.9 | 48 | 12.7 | 59 | 15.1 | 1.34 (1.05-1.70) | 1.22 (0.81-1.84) |
| Bipolar and Related Disorders | 157 | 4.6 | 87.0 | 3.2 | 70 | 9.1 | 24 | 6.3 | 46 | 11.8 | 2.97 (2.15-4.12) | 1.97 (1.17-3.29) |
| Communication Disorders | 18 | 0.5 | 14.0 | 0.5 | 4 | 0.5 | 2 | 0.5 | 2 | 0.5 | 0.99 (0.24-3.17) | 0.97 (0.07-13.39) |
| Depressive Disorders | 1309 | 38.1 | 888.0 | 32.9 | 421 | 54.7 | 198 | 52.4 | 223 | 57.0 | 2.43 (2.06-2.86) | 1.21 (0.91-1.60) |
| Developmental Delay or Unspecified Neurodevelopmental Disorder | 67 | 1.9 | 55.0 | 2.0 | 12 | 1.6 | 7 | 1.9 | 5 | 1.3 | 0.75 (0.40-1.42) | 0.69 (0.22-2.18) |
| Disruptive, Impulse Control and Conduct Disorders | 203 | 5.9 | 99.0 | 3.7 | 104 | 13.5 | 41 | 10.8 | 63 | 16.1 | 4.06 (3.05-5.42) | 1.58 (1.04-2.41) |
| Dissociative Disorders | 2 | 0.1 | 0.0 | 0.0 | 2 | 0.3 | 0 | 0.0 | 2 | 0.5 | - | - |
| Elimination Disorders | 9 | 0.3 | 9.0 | 0.3 | 0 | 0.0 | 0 | 0.0 | 0 | 0.0 | - | - |
| Feeding and Eating Disorders | 102 | 3.0 | 86.0 | 3.2 | 16 | 2.1 | 11 | 2.9 | 5 | 1.3 | 0.64 (0.37-1.10) | 0.43 (0.15-1.26) |
| Fetal or Newborn Damage Related to Maternal Substance Abuse | 7 | 0.2 | 7.0 | 0.3 | 0 | 0.0 | 0 | 0.0 | 0 | 0.0 | - | - |
| Intellectual Disability | 57 | 1.7 | 46.0 | 1.7 | 11 | 1.4 | 2 | 0.5 | 9 | 2.3 | 0.83 (0.43-1.61) | 4.43 (0.95-20.64) |
| Maternal Mental Illness or Substance Abuse During Preg, Delivery or Post Partum | 3 | 0.1 | 1.0 | 0.0 | 2 | 0.3 | 1 | 0.3 | 1 | 0.3 | - | - |
| Mental Health Symptom | 454 | 13.2 | 256.0 | 9.5 | 198 | 25.7 | 83 | 22.0 | 115 | 29.4 | 3.27 (2.66-4.02) | 1.48 (1.07-2.05) |
| Miscellaneous | 170 | 4.9 | 135.0 | 5.0 | 35 | 4.6 | 17 | 4.5 | 18 | 4.6 | 0.90 (0.61-1.31) | 1.02 (0.52-2.02) |
| Motor Disorders | 38 | 1.1 | 25.0 | 0.9 | 13 | 1.7 | 8 | 2.1 | 5 | 1.3 | 1.82 (0.93-3.57) | 0.60 (0.19-1.85) |
| Neurocognitive Disorders | 61 | 1.8 | 59.0 | 2.2 | 2 | 0.3 | 0 | 0.0 | 2 | 0.5 | 0.11 (0.01-0.44) | - |
| Obsessive-Compulsive and Related Disorders | 160 | 4.7 | 113.0 | 4.2 | 47 | 6.1 | 21 | 5.6 | 26 | 6.6 | 1.47 (1.04-2.09) | 1.21 (0.67-2.19) |
| Personality Disorders | 23 | 0.7 | 17.0 | 0.6 | 6 | 0.8 | 2 | 0.5 | 4 | 1.0 | 1.23 (0.48-3.12) | 1.94 (0.35-10.67) |
| Schizophrenia Spectrum and Other Psychotic Disorders | 130 | 3.8 | 77.0 | 2.9 | 53 | 6.9 | 15 | 4.0 | 38 | 9.7 | 2.49 (1.74-3.57) | 2.61 (1.41-4.82) |
| Sexuality and Gender Identity Disorders | 33 | 1.0 | 19.0 | 0.7 | 14 | 1.8 | 6 | 1.6 | 8 | 2.0 | 2.59 (1.29-5.19) | 1.30 (0.45-3.77) |
| Sleep-Wake Disorders | 1 | 0.0 | 1.0 | 0.0 | 0 | 0.0 | 0 | 0.0 | 0 | 0.0 | - | - |
| Somatic Symptom and Related Disorders | 24 | 0.7 | 23.0 | 0.9 | 1 | 0.1 | 0 | 0.0 | 1 | 0.3 | 0.15 (0.004-0.93) | - |
| Specific Learning Disorders | 12 | 0.3 | 8.0 | 0.3 | 4 | 0.5 | 2 | 0.5 | 2 | 0.5 | 1.74 (0.52-5.80) | 0.97 (0.14-6.90) |
| Substance Abuse-Related Medical Illness | 0 | 0.0 | 0.0 | 0.0 | 0 | 0.0 | 0 | 0.0 | 0 | 0.0 | - | - |
| Substance Related and Addictive Disorders | 458 | 13.3 | 356.0 | 13.2 | 102 | 13.3 | 38 | 10.1 | 64 | 16.4 | 0.99 (0.79-1.26) | 1.75 (1.14-2.69) |
| Suicide or Self-Injury | 1170 | 34.0 | 715.0 | 26.5 | 455 | 59.2 | 192 | 50.8 | 263 | 67.3 | 3.96 (3.35-4.69) | 1.99 (1.49-2.67) |
| Trauma and Stressor-Related Disorders | 209 | 6.1 | 127.0 | 4.7 | 82 | 10.7 | 34 | 9.0 | 48 | 12.3 | 2.39 (1.79-3.20) | 1.42 (0.89-2.25) |
| Psychiatric Comorbidity (≥ 2 CAMHD-CS diagnostic groups) | 2042 | 59.4 | 1368.0 | 50.6 | 674 | 87.6 | 304 | 80.4 | 370 | 94.6 | 6.76 (5.38-8.49) | 4.29 (2.58-7.13) |
| Acute Care Use |  |  |  |  |  |  |  |  |  |  |  |  |
| Prior ED use (≥ 1 visit) |  |  |  |  |  |  |  |  |  |  |  |  |
| Past 90 days | 546 | 15.9 | 399 | 14.8 | 147 | 19.1 | 81 | 21.4 | 66 | 16.9 | 1.30 (1.03-1.64) | 1.83 (1.37-2.45) |
| Past 180 days | 753 | 21.9 | 543 | 20.1 | 210 | 27.3 | 120 | 31.7 | 90 | 23.0 | 1.45 (1.17-1.79) | 0.85 (0.57-1.27) |
| Past 365 days | 1004 | 29.2 | 742 | 27.5 | 262 | 34.1 | 150 | 39.7 | 112 | 28.6 | 1.26 (1.02-1.55) | 0.77 (0.53-1.11) |
| Prior Medical Hospitalization (≥ 1 visit) |  |  |  |  |  |  |  |  |  |  |  |  |
| Past 90 days | 74 | 2.2 | 72 | 2.7 | 2 | 0.3 | 1 | 0.3 | 1 | 0.3 | 0.11 (0.03-0.46) | 0.72 (0.50-1.03) |
| Past 180 days | 113 | 3.3 | 107 | 4.0 | 6 | 0.8 | 4 | 1.1 | 2 | 0.5 | 0.22 (0.09-0.53) | 0.64 (0.11-3.86) |
| Past 365 days | 174 | 5.1 | 159 | 5.9 | 15 | 2.0 | 11 | 2.9 | 4 | 1.0 | 0.38 (0.21-0.70) | 0.47 (0.14-1.59) |
| Prior Psychiatric Hospitalization (≥ 1 visit) |  |  |  |  |  |  |  |  |  |  |  |  |
| Past 90 days | 116 | 3.4 | 45 | 1.7 | 71 | 9.2 | 38 | 10.1 | 33 | 8.4 | 5.73 (3.85-8.54) | 0.74 (0.44-1.24) |
| Past 180 days | 184 | 5.3 | 67 | 2.5 | 117 | 15.2 | 67 | 17.7 | 50 | 12.8 | 6.72 (4.83-9.36) | 0.65 (0.42-0.99) |
| Past 365 days | 242 | 7.0 | 91 | 3.4 | 151 | 19.6 | 87 | 23.0 | 64 | 16.4 | 5.72 (4.24-7.72) | 0.56 (0.37-0.85) |
| Medications received during ED visit |  |  |  |  |  |  |  |  |  |  |  |  |
| Antidepressant | 689 | 20.0 | 447 | 16.5 | 242 | 32.7 | 66 | 17.5 | 176 | 45.0 | 2.82 (1.90-2.74) | 3.87 (2.78-5.39) |
| Antiepileptic | 153 | 4.4 | 87 | 3.2 | 66 | 8.9 | 16 | 4.2 | 50 | 12.8 | 2.79 (2.00-3.88) | 3.32 (1.85-5.94) |
| Antihistamine | 127 | 3.7 | 85 | 3.1 | 42 | 5.7 | 10 | 2.6 | 32 | 8.2 | 1.76 (1.20-2.57) | 3.28 (1.5-6.77) |
| Antipsychotic | 458 | 13.3 | 257 | 9.5 | 201 | 27.2 | 60 | 15.9 | 141 | 36.1 | 3.32 (2.71-4.08) | 2.99 (2.12-4.22) |
| Anxiolytic | 295 | 8.6 | 206 | 7.6 | 89 | 12.0 | 23 | 6.1 | 66 | 16.9 | 1.57 (1.20-2.40) | 3.13 (1.91-5.16) |
| Hypnotic or Sedative | 62 | 1.8 | 43 | 1.6 | 19 | 2.6 | 6 | 1.6 | 13 | 3.3 | 1.55 (0.90-2.67) | 2.13 (0.80-5.67) |
| Lithium | 52 | 1.5 | 27 | 1.0 | 25 | 3.4 | 11 | 2.9 | 14 | 3.6 | 3.29 (1.90-5.70) | 1.24 (0.56-2.76) |
| Psychostimulant | 273 | 7.9 | 184 | 6.8 | 29 | 3.9 | 23 | 6.1 | 55 | 14.1 | 1.77 (1.35-2.31) | 3.13 (1.91-5.16) |
| Injectable medication | 110 | 3.2 | 68 | 2.5 | 42 | 5.7 | 6 | 1.6 | 36 | 9.2 | 2.21 (1.49-3.28) | 6.29 (2.62-15.10) |
| Urine Drug Screen Results (Positive)^c^ |  |  |  |  |  |  |  |  |  |  |  |  |
| Amphetamine | 71 | 2.1 | 36 | 1.3 | 35 | 4.7 | 12 | 3.2 | 23 | 5.9 | 1.17 (0.72-1.90) | 0.56 (0.27-1.18) |
| Benzodiazepines | 47 | 1.4 | 23 | 0.9 | 24 | 3.2 | 7 | 1.9 | 17 | 4.3 | 1.26 (0.70-2.26) | 0.74 (0.30-1.85) |
| Cannabis | 199 | 5.8 | 109 | 4.0 | 90 | 12.2 | 25 | 6.6 | 65 | 16.6 | 0.98 (0.71-1.34) | 0.76 (0.45-1.29) |
| Cocaine | 9 | 0.3 | 6 | 0.2 | 3 | 0.4 | 1 | 0.3 | 2 | 0.5 | 0.59 (0.09-2.80) | 0.62 (0.03-37.01) |
| Opiates | 9 | 0.3 | 6 | 0.2 | 3 | 0.4 | 1 | 0.3 | 2 | 0.5 | 0.59 (0.09-2.80) | 0.62 (0.03-37.01) |
| Ethanol | 42 | 1.2 | 34 | 1.3 | 8 | 1.1 | 1 | 0.3 | 7 | 1.8 | 0.27 (0.12-0.58) | 2.21 (0.27-18.19) |
| Disposition |  |  |  |  |  |  |  |  |  |  |  |  |
| Discharged without hospitalization^d^ | 2127 | 61.8 | 1801 | 66.7 | 326 | 42.4 | 245 | 64.8 | 81 | 20.7 | 0.37 (0.31-0.43) | 0.15 (0.11-0.21) |
| General medical hospitalization^e^ | 315 | 9.2 | 307 | 11.4 | 8 | 1.0 | 4 | 1.1 | 4 | 1.0 | 0.15 (0.09-0.25) | 0.97 (0.18-5.22) |
| Psychiatric hospitalization | 937 | 27.2 | 508 | 18.8 | 429 | 55.8 | 129 | 34.1 | 300 | 76.7 | 3.53 (2.96-4.22) | 1.75 (1.30-2.34) |
| Within health system | 706 | 20.5 | 430 | 15.9 | 276 | 35.9 | 118 | 31.2 | 158 | 40.4 | 2.92 (2.44-3.49) | 1.49 (1.11-2.01) |
| Transferred outside health system | 198 | 5.8 | 62 | 2.3 | 136 | 17.7 | 5 | 1.3 | 131 | 33.5 | 9.04 (6.61-12.36) | 37.59 (15.17-93.10) |
| *Note: ^a^Other race and ethnicity includes patient refused and unknown; ^b^Diagnostic code groups from Child and Adolescent Mental Health Disorders Classification System (CAMHD-CS), ^c^Urine drug screen was obtained for 27.5% sample.. ^c^Discharged without hospitalization includes eloped (n=4), left without being seen (2), left against medical advice (2), inpatient rehab facility (3), law enforcement (1), skilled nursing (1), expired (3);Odds ratios omitted when sample size was insufficient. 95% Confidence Intervals calculated via Fisher Exact Tests for 2x2 matrices with N < 5 in any cell.* | | | | | | | | | | | | |

| **Table S2.** Classifier performance metrics and contingency matrix for detainment use and continuation. | | | | | | | |
| --- | --- | --- | --- | --- | --- | --- | --- |
|  | | | | | **Random Forest Classifier** | | |
|  | **TP** | **FP** | **FN** | **TN** | **Total** | **Accuracy (95% CI)** | **AUC-ROC (95% CI)** |
| Detainment use | 304 | 235 | 465 | 2436 | 2702 | 0.796 (0.783-0.809) | 0.820 (0.807-0.820) |
| Detainment continuation | 278 | 102 | 113 | 276 | 769 | 0.720 (0.689-0.752) | 0.803 (0.883-0.775) |
